# Imaging technologies from bench to bedside

**DOI:** 10.1186/s12967-015-0449-5

**Published:** 2015-03-21

**Authors:** Ravinder Reddy, Mohammad Haris

**Affiliations:** Center for Magnetic Resonance and Optical Imaging, Perelman School of Medicine, Department of Radiology, University of Pennsylvania, 422 Curie Boulevard, Philadelphia, PA 19104-6100 USA; Research Branch, Sidra Medical and Research Center, Doha, Qatar

The last few decades have seen tremendous advances in medicine that have enhanced understanding of pathophysiological processes at the cellular and molecular level, and led to the development of increasingly sophisticated diagnostic imaging technologies. Early detection of disease induced molecular and functional changes before induction of irreversible structural changes is key for optimal treatment efficacy. Non-invasive imaging modalities, such as positron emission tomography (PET) [1], single photon emission computed tomography (SPECT) [2], computed tomography (CT) [3], optical tomographic technologies [4], magnetic resonance imaging (MRI) [5], ultrasound (US) [6], and X-rays play a vital role in both the diagnosis and monitoring of disease in response to therapy. These techniques cover a broad range of spatio-temporal resolution and varying degrees of sensitivity and specificity to different molecular changes, and in many cases provide complementary information [7,8]. Recently discovered molecular targets of various disease states, including oncology, neurodegenerative and neuropsychiatric, cardiovascular, and musculoskeletal pathologies, drive further developments in the imaging field to detect these new molecular markers. Ultimately, these technologies contribute to improved disease management and personalized patient care.

Standard-of-care medical imaging techniques such as X-rays, US, CT and MRI provide exquisite structural details of human anatomy. These methods are the first-line techniques in clinic for diagnosis and characterization of disease, based primarily on structure/morphology such as size, texture and tissue attenuation [8]. In addition to providing diagnostic information, the US modality has the additional benefit of use as a therapeutic tool [6,7,9].

Functional nuclear medicine techniques (PET and SPECT) provide a unique, non-invasive assessment of intracellular processes and enzyme trafficking, receptors and gene expression, and serve as the underpinnings of molecular medicine. These techniques provide non-invasive diagnostic information about biochemical and physiological process ranging from glucose metabolism to gene expression by evaluating the kinetics of short-lived radioisotope tracers. While many promising tracers have been synthesized that target a variety of metabolic pathways or specific markers ^18^F-fluorodeoxyglucose (FDG), a glucose analogue is the main radiotracer in clinical practice today. In addition, these functional nuclear medicine techniques are also being used in research and clinical settings to detect and evaluate Alzheimer’s disease, metabolic viability of cardiac tissues, *in vivo* gene expression, and in tracking of cancer metastasis to different organs [7,8,10-12].

MRI is one of the most powerful and versatile non-invasive techniques. The major advantage of MRI is that it provides high-resolution, three-dimensional images of tissue structure, as well as functional and metabolic information. Furthermore, MRI is performed *in vivo* without the use of any ionizing radiation, allowing for repeated study. Several advanced MRI methods have been introduced to monitor the structural [13], functional [14,15] as well as biochemical changes in various diseases. Magnetic resonance spectroscopy (MRS), which provides the information about the biochemical signatures, is an additional important clinical research tool to assess and characterize disease pathophysiology [16,17].

Using MRS, enriched metabolites (e.g. ^13^C enriched) can be used to probe endogenous reaction kinetics. Latest advances in chemical exchange saturation transfer (CEST) MRI show promise in detecting several endogenous metabolites and proteins with substantially enhanced sensitivity (at least an order of magnitude) compared to conventional MRS [18-25]. Recent developments in hyperpolarized imaging based on dynamic nuclear polarization (DNP) of ^13^C enriched pyruvate are yielding highly promising preclinical results [26,27] exploring *in vivo* reactions in oncology and other disease conditions. Some very preliminary results showing the promise of these methods in addressing clinical problems in patients have been demonstrated [28].

Optical imaging is another emerging imaging modality with high potential for improving diseases diagnosis and treatment, which can be readily set up at the patient’s bedside or in the operating room [4,29-31]. Optical imaging uses non-ionizing radiation and offers potentially to image organs, tissues as well as smaller structures including cells and molecules using their unique photon absorption or scattering profiles. It also differentiates between native soft tissue and tissue labeled with endogenous or exogenous probes based on their wavelength dependent photon absorption or scattering pattern [32-35]. Despite limitations in their spatial resolution, optical imaging methods offer capabilities for studying functional and molecular events in different pathophysiological conditions. There are several techniques in optical imaging that are currently being used both in research and clinical setting for evaluating various diseases and therapeutic responses [30,36-38]. Potentially, optical imaging can also be combined with other imaging modalities to improve the patient’s clinical management.

PET, SPECT and near-infrared reflectance fluorescence optical imaging techniques have relatively high sensitivity and can detect compounds with concentrations in micro- to pico-molar range [7]. Despite the high sensitivity these methods are beset by a relatively low spatial resolution (5 to 10 mm in clinical setting). Also, in many cases the emitting ligands may lose the specificity. One issue with nuclear medicine techniques is the use of nuclear radiation, which precludes their repeat use in short time spans. On the other hand, MRI provides high spatial resolution (in hundreds of micrometers range), but is relatively insensitive, in comparison to nuclear medicine techniques mentioned above; it requires concentrations of metabolites to be detected to be in the millimolar range and few endogenous molecules or metabolites can be imaged [39].

A milestone in the field of diagnostic imaging is the emergence of integrated structural and functional modalities such as combined PET-CT and PET-MRI [40,41]. These integrated modalities provide concurrent structural, molecular and functional information, improve the multimodal imaging correlations and ease the patient burden for multiple imaging sessions.

Combining these advanced imaging techniques will result in improved precision of the data that are intrinsically more sensitive to the underlying pathophysiology than the morphological features available in routine structural imaging. Over the years, all these powerful imaging techniques have been improving the way the diseases are diagnosed, therapeutic responses are monitored and dramatically enhancing the practice of medicine making it more prognostic, preventative and personalized. Despite these advances, many technological innovations in these imaging modalities are still in research setting. Transferring these technologies into clinical setting requires an intense collaborative effort between researchers in imaging physics, instrumentation, image processing, biologists, chemists, and regulatory bodies as well as clinicians from all branches of medicine. This journal section facilitates communication of advances in translating the imaging modalities from mere research tools to clinical setting. We welcome research articles from all the stakeholders in this field.
